# Pharmacotherapeutic considerations of selective estrogen receptor modulators for vascular protection

**DOI:** 10.3389/fphar.2026.1749904

**Published:** 2026-01-15

**Authors:** Janette Al Banna, Farah Karam, Dalia Hassanieh, Youssuf H. Khanafer, Mohammed Seed Ahmed, Hussein Sharara, Ali H. Eid

**Affiliations:** 1 Faculty of Medicine and Medical Sciences, University of Balamand, Balamand, Lebanon; 2 College of Medicine, QU Health, Qatar University, Doha, Qatar; 3 Department of Basic Medical Sciences, College of Medicine, QU Health, Qatar University, Doha, Qatar; 4 Neuroscience Research Center, Mashhad University of Medical Sciences, Mashhad, Iran

**Keywords:** cardiovascular disease, estrogen receptors, postmenopausal health, precision therapeutics, SERM, vascular pharmacology

## Abstract

Selective estrogen receptor modulators (SERMs) are nonsteroidal compounds that exert context-dependent agonist or antagonist effects on estrogen receptors through ligand-induced conformational changes that govern coactivator or corepressor recruitment. This biochemical selectivity underlies their tissue-specific pharmacological actions. In the vasculature, SERMs modulate endothelial nitric oxide synthase (eNOS) activity, attenuate vascular smooth muscle cell (VSMC) proliferation, and regulate oxidative stress pathways, while also influencing platelet reactivity through NADPH oxidase–dependent mechanisms. Among the most studied SERMs are Tamoxifen and Raloxifene. Tamoxifen functions as a prodrug, requiring hepatic bioactivation, primarily by CYP2D6 and CYP3A4, to form active metabolites, notably 4-hydroxytamoxifen and endoxifen, with enhanced receptor affinity. In contrast, raloxifene undergoes extensive glucuronidation, resulting in low systemic bioavailability of the active compound. However, the systemic concentrations achieved are sufficient to confer measurable vascular effects. Despite these pharmacokinetic differences, both agents improve lipid and fibrinogen profiles, but also increase venous thromboembolism risk through modulation of coagulation pathways. Clinical trials confirm benefits in oncology and bone health, yet fail to demonstrate consistent reductions in cardiovascular endpoints. The pharmacological profile of SERMs therefore reflects a delicate equilibrium between receptor-mediated vascular protection and thrombotic liability. Indeed, their raison d’être increasingly extends beyond oncology into cardiovascular endocrine pharmacology, where they serve as prototypes for designing next-generation agents with optimized receptor selectivity and safer vascular outcomes.

## 1 Introduction

Cardiovascular disease (CVD) is the leading cause of death worldwide, accounting for nearly 40% of deaths among individuals over 65 years of age (World Health Organization, 2024). Its burden has risen dramatically over recent decades, with global prevalence nearly doubling between 1990 and 2019, increasing from 271 million to 523 million cases and resulting in 19.1 million deaths in 2020 (Martin et al., 2025). This rise reflects complex interactions between demographic transitions, including population aging, lifestyle changes, accumulation of comorbidities, and medical advances, that while helpful, also heighten long-term cardiovascular health (Zhao et al., 2024). Importantly, epidemiologic data show that CVD incidence varies not only by age and sex, but also by menopausal status. According to the American Heart Association, nearly 40% of adults aged 40–59 years, 75% of those aged 60–79, and 86% of those over 80 are affected by some form of CVD (Zhang et al., 2024). Before menopause, men exhibit nearly threefold higher prevalence compared to women, but this gap narrows sharply after menopause, suggesting a cardiovascular-protective role of estrogen and its receptors (Davezac et al., 2021; Ahmed and Abdel-Rahman, 2025). These observations highlight the significance of estrogen signaling pathways in shaping sex-specific differences in CVD (Wehbe Z. et al., 2020; Fardoun M. et al., 2020; Dehaini et al., 2018).

Pharmacological agents that influence estrogen pathways represent a particularly important factor in this context, with SERMs serving as a prominent example. Originally developed for reproductive and oncologic applications, SERMs have been widely prescribed for breast cancer prevention, osteoporosis treatment and other gynecologic conditions (Matsushima-Nish et al., 2022). However, their effects extend beyond reproductive tissues, as they engage estrogen receptors (ERs) across metabolic and cardiovascular systems. This duality explains their capacity to produce beneficial outcomes, such as favorable modulation of lipid profiles and vascular tones, while simultaneously elevating thromboembolic risk under certain conditions (Ewer and Gluck, 2009). Given these contrasting actions, therapeutic decisions regarding SERM use require careful balancing of reproductive benefits against cardiovascular implications. The following sections therefore first examine the physiology of estrogen and its receptors, establishing the foundation for understanding how SERMs modulate cardiovascular health.

Accordingly, the central aim of this review is to critically examine how SERMs modulate vascular biology and cardiovascular risk through estrogen receptor–dependent mechanisms. Rather than positioning SERMs as primary cardioprotective therapies, this article specifically evaluates their molecular, cellular, and clinical vascular effects—highlighting both protective actions (e.g., endothelial function, lipid modulation) and adverse liabilities (e.g., thrombosis). By integrating mechanistic insights with evidence from major clinical trials, this review seeks to clarify why favorable surrogate vascular markers have not consistently translated into improved cardiovascular outcomes, and to define the implications of these findings for the future design and clinical use of next-generation estrogen receptor–targeted therapies.

## 2 Estrogen and cardiovascular health

### 2.1 Estrogen and its primary site of production

Estrogens are a family of steroidal sex hormones that regulate reproductive organ function in both sexes, while also governing diverse systemic processes relevant to cardiovascular physiology (Toporcer et al., 2024; Abdollahpour et al., 2025). Four major estrogens—estrone (E1), estradiol (E2), estriol (E3), and estretrol (E4)—exhibit distinct roles and secretion patterns across different physiological states. E1 predominates after menopause, while E3 and E4 are produced almost exclusively during pregnancy (Gregory et al., 2025; Gerard and Foidart, 2023). E2, also known as 17β-estradiol, is the most potent and biologically abundant estrogen in premenopausal women and is often considered the principal form in studies of cardiovascular regulation (Coelingh Bennink et al., 2017; Fardoun M. M. et al., 2020; Eid et al., 2007). Because of its dominant role outside pregnancy, estradiol serves as the focal point for understanding estrogen-mediated cardiovascular effects.

Estrogen is synthesized in both gonadal and extra-gonadal tissues, contributing to systemic and local effects across various organs (Coelingh Bennink et al., 2017). In females of reproductive age, the ovaries constitute the primary site of estrogen production, with biosynthesis initiated in the theca cells and completed in granulosa cells through enzymatic conversion (Barakat et al., 2016; Eissa and Gohar, 2023). In males, the testes produce smaller quantities of estrogen, underscoring the hormone’s relevance beyond female reproduction (Eissa and Gohar, 2023; Fardoun et al., 2024; Fardoun et al., 2022). Extra-gonadal sources, particularly adipose tissue, represent a significant source of estrogen for both sexes, becoming more prominent with age and increased fat mass (Barakat et al., 2016; Hetemaki et al., 2021). Additional extra-gonadal sites of estrogen synthesis include bone, adrenal glands, skin, muscle, vascular endothelium, intestines, liver, and vascular smooth muscle. In these tissues, estrogen acts locally through paracrine or intracrine signaling rather than entering systemic circulation (Barakat et al., 2016; Eissa and Gohar, 2023; Iorga et al., 2017). Such extra-gonadal activity becomes the predominant source of estrogen production in postmenopausal women and in men, highlighting its significance once ovarian and testicular output declines (Lee and Den Hartigh, 2025; Hetemaki et al., 2025).

### 2.2 Estrogen receptors and their location

Estrogen exerts its multifaceted biological effects primarily through binding to well-characterized receptor subtypes located across various target tissues (Bolt et al., 2024). The estrogen receptor (ER) family comprises two canonical nuclear receptors, estrogen receptor-α (ERα) and estrogen receptor-β (ERβ) as well as the membrane-associated G-protein coupled receptor known as GPR30 (GPER1) (Bopassa et al., 2010; Hojnik et al., 2023). Each contains distinct functional domains enabling DNA-binding, ligand-binding, and transcriptional activation (Makar et al., 2020). Upon estrogen binding, Estrogen primarily acts through two classical nuclear receptors. Once bound to these receptors, ERα and ERβ undergo conformational changes that facilitate dimerization and translocation to the nucleus, where they regulate gene expression via direct binding to estrogen response elements, or via modulating other transcription factors, thereby initiating the classical genomic pathways (Aryan et al., 2020; Irani et al., 2024). Complementing this, membrane-associated ERs and GPR30 mediate rapid non-genomic pathway. Estrogen engages membrane-associated ERα, ERβ, or GPR30, activating rapid signaling by activating intracellular kinase pathways, such as MAPK and PI3K, which influence gene expression and cellular responses within minutes (Hojnik et al., 2023; Aryan et al., 2020). This duality of genomic and non-genomic pathways enables estrogen to exert both immediate and sustained effects.

The tissue-specific distribution of ERs critically shapes the hormone’s biological actions (Jiang et al., 2023). ERα is predominantly expressed in reproductive tissues, including the mammary glands, uterus, ovarian theca cells, as well as in the liver, adipose tissue, kidney, and bones (Yu et al., 2020). Functionally, it drives reproductive development, metabolic, regulation, and skeletal maintenance (Gregory et al., 2025). In contrast, ERβ is more abundant in the prostate epithelium, ovarian granulosa cells, bone marrow, colon, immune cells and brain, often acting as a modulator by forming heterodimers with ERα to attenuate its transcriptional activity (Gregory et al., 2025). GPR30 is also widely expressed, particularly in reproductive organs, brain, adrenal glands, adipocytes, bone, kidney, and heart, whereby it mediates rapid, non-genomic signaling that influences vascular tone, glucose metabolism, and neuronal activity (Eissa and Gohar, 2023; Wang et al., 2017).

Within the cardiovascular system, both ERα and ERβ are expressed in cardiomyocytes, vascular smooth muscle cells (VSMCs), and endothelial cells, where they regulate nitric oxide (NO) production, vascular remodeling, and cardiac responses to hemodynamic stress (Davezac et al., 2021; Iorga et al., 2017). Importantly, mechanisms of crosstalk between these signaling pathways converge on transcriptional regulation, whereby phosphorylation events mediated by GPR30-activated kinases enhance the transcriptional activity of nuclear receptors and associated transcription factors such as AP-1, STATs, and NF-κB (Fuentes and Silveyra, 2019). This integrated signaling network allows estrogen to exert finely tuned, context-dependent effects essential for cardiovascular function and systemic endocrine balance (Fuentes and Silveyra, 2019). Understanding these complex receptor mechanisms provides critical insights for therapeutic targeting of estrogen signaling in diverse diseases affecting reproductive, metabolic, and cardiovascular systems. Importantly, this heterogeneous distribution and signaling integration of ERα, ERβ, and GPR30 within the cardiovascular system establishes the mechanistic basis by which selective estrogen receptor modulation can yield divergent vascular outcomes—ranging from endothelial protection to maladaptive remodeling—depending on receptor subtype engagement, tissue context, and hormonal milieu.

### 2.3 Variation of Estrogen with gender, age and in relation to menopause

Gender, age, and menopausal status are critical determinants of circulating estrogen levels and, consequently, have profound implications on cardiovascular health. In men, plasma estrogen concentrations remain relatively stable across adulthood, and begin to decline gradually after the age of 60 (Davezac et al., 2021). Contrastingly, premenopausal women exhibit significantly higher serum estrogen levels, generally ranging from 15 to 300 pg/mL during the reproductive years. However, following menopause, this concentration sharply declines to less than 15–40 pg/mL, resulting in postmenopausal estrogen levels comparable to those in age-matched men (Davezac et al., 2021; Richardson et al., 2020; Chadid et al., 2019). This sharp reduction in estrogen following menopause is associated with a markedly increased risk of CVD. Indeed, studies indicate a 4.3-fold higher incidence of CVD in postmenopausal women compared to men in the same age bracket (El Khoudary et al., 2020).

In addition to reduced estrogen levels, menopause is associated with decreased expression and altered signaling of ERs, which further exacerbates cardiovascular vulnerability (Davezac et al., 2021). The expression of ERα, the principal receptor mediating estrogen’s effects in endothelial cells, declines by roughly 33% after menopause, compared to levels during the late follicular phase of the menstrual cycle (Gavin et al., 2009). This decline is largely attributed to reduced estrogen availability (Gavin et al., 2009). Aging also contributes to this decrease in ERα expression with transcriptional downregulation observed in aging tissues (Novensa et al., 2011). Notably, this effect appears sex specific as ERα expression remains stable in male murine models (Zhang et al., 2024). Therefore, these findings underscore that decreased estrogen availability plays a crucial role in influencing receptor expression, whereas aging may further modulate this process in a context-dependent and sex-specific manner. Collectively, the interplay of declining estrogen and receptor changes during menopause shapes cardiovascular outcomes and highlights the need for targeted interventions in this population.

### 2.4 Cardioprotective effect of Estrogen

Gender significantly influences CVD prevalence, with premenopausal women exhibiting relative cardiovascular-protection compared to men—a notion largely attributed to estrogen and its receptors (Davezac et al., 2021). While extensive research has elucidated many estrogen-mediated cardiovascular benefits, the precise mechanisms by which specific ERs subtypes confer protection remain incompletely understood (Aryan et al., 2020; Menazza and Murphy, 2016). Estrogen exerts direct effects on cardiovascular system by promoting vasorelaxation, as well as enhancing proliferation and migration of vascular endothelial cells, thus facilitating vascular repair and health. In contrast, it suppresses proliferation and migration of VSMCs, thereby mitigating vascular remodeling. Estrogen also modulates cardiomyocyte function by improving insulin sensitivity, and protecting against myocardial infarction, cardiac hypertrophy, and ischemia–reperfusion injury through complex genomic and non-genomic signaling. A key component of estrogen’s cardioprotective action includes GPR30-mediated reduction in low-density lipoprotein cholesterol (LDL-C) transcytosis across endothelial cells, a crucial early event in atherogenesis (Eissa and Gohar, 2023; Iorga et al., 2017; Menazza and Murphy, 2016). This mechanism highlights a novel endothelial-specific pathway by which estrogen lowers LDL-C entry into vascular walls, further contributing to cardiovascular protection (Sessa, 2018). These multifaceted mechanisms collectively underpin the observed gender differences in CVD incidence during reproductive years, emphasizing estrogen’s pivotal role in cardiovascular protection.

In addition to these direct actions, estrogen indirectly promotes cardiovascular health by modulating coagulation factors, reducing oxidative stress through decreased production of reactive oxygen species (ROS), and favorably influencing metabolic processes (Eissa and Gohar, 2023). However, these beneficial effects decline with advancing age, particularly after the late postmenopausal phase (above 59 years), coinciding with increased cardiovascular risk (Menazza and Murphy, 2016). This attenuation is linked to age-related alterations in ER expression and signaling efficiency, diminishing estrogen’s protective capacity (Gurrala et al., 2021). Recognizing this critical transition is essential for developing targeted strategies to sustain cardiovascular health in aging women.

## 3 SERMs

### 3.1 Introducing SERMs

SERMs are nonsteroidal compounds that exert estrogenic or antiestrogenic effects in a tissue-selective manner (Linowiecka et al., 2024). This dual functionality enables SERMs to act as agonists in some tissues while serving as antagonists in others, thereby expanding their clinical utility in managing postmenopausal osteoporosis, hormone-responsive cancers, and CVD (Hetemaki et al., 2021; Haider et al., 2024). Developed since 1960s, SERMs such as tamoxifen—the first to gain FDA approval—transformed the therapeutic landscape for estrogen-sensitive conditions (Howell and Howell, 2023). Indeed, tamoxifen not only treats metastatic breast cancer but also serves as a chemo-preventive agent for high-risk women (Manna et al., 2023). Following tamoxifen, subsequent generations like raloxifene and bazedoxifene expanded indications to include osteoporosis prevention and mitigation of menopausal vasomotor symptoms (Kulkarni et al., 2025; Shin et al., 2024), illustrating ongoing pharmaceutical innovation to refine efficacy and safety.

Tamoxifen initial identification as an antifertility agent in animal models preceded its clinical approval in 1977 for metastatic breast cancer (Iorga et al., 2017). In 1998, it gained further FDA approval as a chemo-preventive drug demonstrating efficacy in lowering breast cancer risk among high-risk populations (Mansour et al., 2025; Ren et al., 2025). Similarly, raloxifene underwent rigorous clinical evaluation with FDA approval in 1998 for osteoporosis prevention and treatment, followed by approval in 2007 for breast cancer risk reduction (Maximov et al., 2013). Bazedoxifene, a third-generation SERM, received FDA approval in 2013 for treating vasomotor symptoms and preventing postmenopausal osteoporosis (Mirkin et al., 2014). Moreover, numerous novel SERMs with unique pharmacological profiles have been developed over recent decades to address diverse therapeutic needs, extending the clinical armamentarium against estrogen-responsive diseases.

The advent of SERMs marked a turning point in gynecologic oncology and bone health management. Tamoxifen and raloxifene continue as pivotal treatments for ER-positive breast cancer and osteoporosis prevention, with robust evidence supporting their efficacy in maintaining favorable lipid profiles and reducing breast cancer incidence among high-risk women (Peng et al., 2009; Martinkovich et al., 2014). Clinical trials further substantiate that raloxifene and bazedoxifene significantly reduce vertebral fractures in postmenopausal women, reinforcing their role in bone health (Duggan and McKeage, 2011). Beyond bone and cancer protection, SERMs have been investigated for contraception, ovulatory dysfunction, and even improvements in skin aging (Makar et al., 2020; Aryan et al., 2020). Overall, these benefits underscore the broad impact of SERMs on gynecologic health and highlight their potential for wider therapeutic use. Continued research into optimizing tissue selectivity and minimizing adverse effects is crucial to fully realize their clinical potential.

The molecular basis of SERMs’ tissue selectivity lies in their differential affinity for ERα and ERβ (Makar et al., 2020). This binding initiates complex intracellular signaling involving recruitment of coactivators and corepressors that ultimately dictate transcriptional, and consequently cellular, responses (Notaro et al., 2023) (Figure 2). Typically, SERMs antagonize ERβ -mediated gene expression at EREs while exhibiting partial agonist activity via ERα (Makar et al., 2020). The presence of GPER1 in ERα-positive breast cancer cells has been shown to alter tamoxifen sensitivity, although the mechanism remains unclear (Zheng and Houston, 2019). This illustrates the complex receptor dynamics underpinning SERM actions. These ligand-receptor interactions and conformational changes govern the distinct agonistic or antagonistic effects observed across tissues, forming the cornerstone of SERM pharmacology.

Despite their unquestioned therapeutic value, the use of SERMs is not without adverse effects, which may indeed limit their clinical use. Common complications include thromboembolic events, osteoporosis, and even carcinogenesis. Findings from the NSABP P-1 trial highlight tamoxifen’s association with increased risks of endometrial cancer, vasomotor symptoms, cataracts, stroke, pulmonary embolism (PE), and drug resistance development (Bolt et al., 2024; Yu et al., 2020; Wang et al., 2017). Conversely, raloxifene displays a more favorable safety profile, lacking increased endometrial cancer risk and reducing breast cancer incidence, as confirmed in the MORE trial (Mansour et al., 2025). The ideal SERM exhibits antagonism in breast and uterine tissues coupled with agonism in bone and cardiovascular systems, maximizing benefit while mitigating risks (Makar et al., 2020). Clinical decision-making must therefore be individualized, carefully balancing therapeutic efficacy and safety to optimize patient outcomes.

### 3.2 Pharmacokinetics and pharmacodynamics of tamoxifen and raloxifene

**FIGURE 1:**
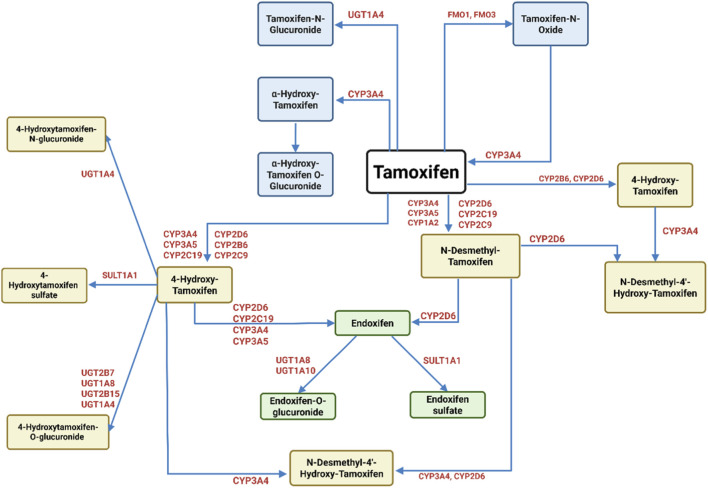
Metabolism of tamoxifen. Tamoxifen undergoes extensive metabolism by cytochrome P450 (CYP) enzymes and phase II enzymes, resulting in both active and inactive metabolites. The primary metabolic pathways include hydroxylation, demethylation, and conjugation. CYP3A4, CYP3A5, CYP2D6, CYP2C9, CYP2C19, and CYP1A2 are involved in generating key intermediates such as 4′-hydroxy-tamoxifen and N-desmethyl-tamoxifen. The latter can be further converted by CYP2D6 into endoxifen, the most potent metabolite with high affinity for ERs. Other pathways lead to the formation of tamoxifen-N-oxide (via FMOs) and glucuronidated or O-glucuronidated metabolites (via UGT1A4), which are generally inactive and excreted. This figure highlights the complex enzymatic interplay dictating the balance between therapeutic efficacy and drug clearance (Sanchez-Spitman et al., 2019; Teunissen et al., 2011).

Pharmacokinetically, tamoxifen (Figure 1) is a lipophilic prodrug extensively metabolized in the liver, predominantly by the cytochrome P450 enzyme CYP2D6, into active metabolites such as 4-hydroxytamoxifen, which exhibits 30 to 100-fold greater potency than the parent molecule (Kanji et al., 2023). This metabolic activation is crucial for tamoxifen’s clinical efficacy, yet *CYP2D6* polymorphisms generate significant inter-individual variability in drug response, especially in ER-positive breast cancer patients (MacLehose et al., 2025). Additional biotransformation by CYP3A4 and other enzymes produce metabolites that circulate systemically and engage target tissues before excretion mainly via feces (Puszkiel et al., 2021). Thus, tamoxifen’s therapeutic response and toxicity are influenced by the patient’s metabolic phenotype.

Mechanistically, tamoxifen induces cancer cell death through multifactorial pathways. It activates caspase-9, inhibits epidermal growth factor receptor signaling, and disrupts mitochondrial function by impeding respiratory complexes I and III and modulating mitochondrial permeability transition pores, promoting apoptosis (Mirzaei et al., 2022; Unten et al., 2022). Tamoxifen also regulates gene expression involved in epithelial-mesenchymal transition, downregulating oncogenic regulators such as *PLK1*, *BIRC5*, and *RACGAP1*, while upregulating tumor suppressor genes including *MYLK*, *SOCS3*, and *STAT5B*, impacting breast cancer metastasis (Mirzaei et al., 2022). Long-term tamoxifen usage perturbs hepatic estrogen homeostasis by downregulating *UGT2B* gene expression, which may contribute to adverse effects like hepatotoxicity (Hao et al., 2022). Cumulatively, tamoxifen initiates mitophagy, necrosis, apoptosis, and autophagy in ER-positive cancer cells via diverse signaling mechanisms.

Raloxifene’s pharmacokinetics contrast markedly with tamoxifen; it exhibits low oral bioavailability (∼2%) due to extensive first-pass metabolism predominantly via glucuronidation by UGT1A2 (Jordan, 2007; Chang et al., 2022; Kushwaha et al., 2013). Alternative delivery routes, such as transdermal, buccal, rectal, and nanoparticle-mediated systems like solid lipid nanoparticles, have shown promise in enhancing systemic exposure (Kushwaha et al., 2013). The major raloxifene metabolites, raloxifene-4′-glucuronide and raloxifene-6-glucuronide, are chiefly eliminated via feces with negligible renal clearance (Du et al., 2021). Raloxifene’s bioavailability and clearance are additionally modulated by factors including age, administration route, and ethnicity.

Raloxifene’s tissue selectivity is primarily driven by structural elements, where the 6-hydroxy substituents confer estrogenic agonist activity and the piperidine side chain mediates antiestrogenic antagonism (Patel et al., 2025). This antagonistic action is achieved through the ligand-receptor interaction that prevents coactivator binding in certain tissues, such as the breast and uterus (Nuttall et al., 2000). Beyond direct ER modulation, raloxifene beneficially influences cardiovascular and metabolic pathways: it enhances endothelial function by increasing the NO to endothelin-1 ratio, reduces plasma homocysteine—an independent cardiovascular risk factor—and inhibits α-glucosidase, suggesting potential in glycemic control (Wu et al., 2022). Furthermore, raloxifene disrupts cholesterol biosynthesis pathways leading to autophagy induction, which may contribute both to its chemotherapeutic effects and metabolic regulatory properties (Chang et al., 2022). These combined actions render raloxifene effective not only for bone and breast protection but also for cardiovascular and metabolic health support.

Despite their clinical efficacy, both tamoxifen and raloxifene face challenges due to acquired drug resistance. This has galvanized research into novel SERMs with enhanced tissue selectivity and safety profiles (Khan et al., 2022). Bazedoxifene, a third-generation SERM, demonstrates efficacy in antiestrogen-resistant breast cancer models, progressing both preclinically and clinically (Tsuji et al., 2022). Moreover, investigational compounds, including naphthalene, anthracenes, benzopyrans, indolones, genistein, indoles, and tetrahydroisoquinolines, are currently undergoing evaluation (Amari et al., 2004). Interestingly, the development of selective estrogen receptor down-regulators also offers emerging alternative or complementary therapeutic options for SERM-resistant malignancies (Bidard et al., 2022). Despite such developments, tamoxifen and raloxifene remain the most utilized SERMs in clinical practice.

**FIGURE 2:**
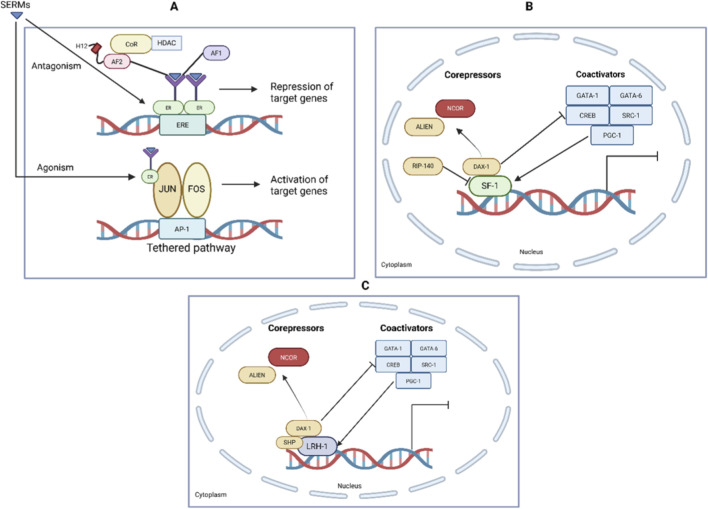
Transcriptional Regulation by SF-1, LRH-1, and SERMs. **(A)** SERMs such as tamoxifen act as ER ligands and can function as agonists or antagonists depending on tissue context. Antagonistic activity involves recruitment of corepressors (CoR, HDAC) to the estrogen response element (ERE), leading to repression of target genes. Agonistic effects occur via tethered pathways, where ERs interact with transcription factors (JUN, FOS at AP-1 sites), promoting gene activation. **(B)** Nuclear receptor SF-1 regulates gene transcription via cofactor recruitment. Corepressors such as NCOR, ALIEN, RIP-140, and DAX-1 inhibit transcription, while coactivators including GATA-1, GATA-6, CREB, SRC-1, and PGC-1 enhance transcriptional activation. **(C)** Similarly, LRH-1 function is regulated by a balance of corepressors (NCOR, ALIEN, DAX-1, SHP) and coactivators (GATA-1, GATA-6, CREB, SRC-1, PGC-1), determining the transcriptional output. This illustrates how SERMs exert tissue-selective actions through modulation of ER and nuclear receptor coregulator complexes (Musa et al., 2007; Meinsohn et al., 2019).

The functional diversity of SERMs is rooted in the dynamic interplay between ligand binding and ER conformational states, which dictate coactivator or corepressor recruitment and subsequent transcriptional activation or repression (Wardell et al. 2014; Fan and Craig, 2014; Baez-Jurado et al., 2019; Komm and Mirkin, 2014) (Figure 2). Tamoxifen and raloxifene predominantly adopt receptor configurations that favor corepressor interaction, thereby inhibiting the formation of the transcription initiation complexes necessary for estrogen response element binding and gene transcription activation (Seo et al., 2023). While the complex landscape of ER modulation remains incompletely understood, ongoing studies continue to unravel the multilayered regulatory mechanisms involving various co-regulators that ultimately determine the tissue-specific and context-dependent effects of SERMs.

Collectively, these pharmacokinetic and mitochondrial-disruptive properties of tamoxifen provide a mechanistic framework through which off-target oxidative stress and altered redox signaling may extend beyond tumor cells to the vasculature, potentially contributing to endothelial dysfunction and increased thrombotic susceptibility observed in susceptible patient populations.

### 3.3 Molecular mechanisms of SERMs effects on cardiovascular physiology

SERMs influence cardiovascular physiology predominantly through their tissue-specific interactions with ERα and ERβ expressed in endothelial and smooth muscle cells. ERα, highly abundant in endothelial cells, is essential for vascular protection, primarily by mitigating angiotensin II–induced hypertension via its transcriptional activity at the AF2 domain (Aryan et al., 2020; Xue et al., 2007). This role is evidenced by worsened hypertensive phenotypes in ERα knockout female mice. Pharmacologic activation with selective ERα agonists such as Cpd1471 restores endothelial nitric oxide synthase (eNOS) expression and endothelial function, underscoring ERα′s pivotal contribution to vascular homeostasis (Favre et al., 2020). In parallel, ERβ attenuates vasoconstriction, reduces vascular resistance, and lowers blood pressure; genetic loss of ERβ disrupts vascular contractility and ion channel function, exacerbating hypertension (Frump et al., 2018). Administration of selective ERβ agonists like 8β-VE2 effectively lowers systolic blood pressure and vascular resistance (Muka et al., 2016). This highlights the complementary and synergistic roles of ERα and ERβ in maintaining vascular function and positioning SERMs as promising agents in hypertensive management.

Beyond classical genomic signaling, SERMs engage in rapid non-genomic pathways (Simoncini et al., 2002). Raloxifene, for example, stimulates NO production via ERα-PI3K interaction, enhancing vascular reactivity and promoting vasodilation (Kang et al., 2022). ERβ activation mitigates vascular inflammation and atherosclerotic progression, while ERα facilitates myocardial glucose uptake (Hulley et al., 1998). In hypertensive models, ERβ upregulation correlates with increased hypoxia-inducible factor-1α (HIF-1α) activity, promoting adaptive vascular remodeling and cardiopulmonary protection (Xie et al., 2022). Collectively, these mechanisms extend SERM benefits beyond cancer prevention to comprehensive cardiometabolic health support.

**FIGURE 3:**
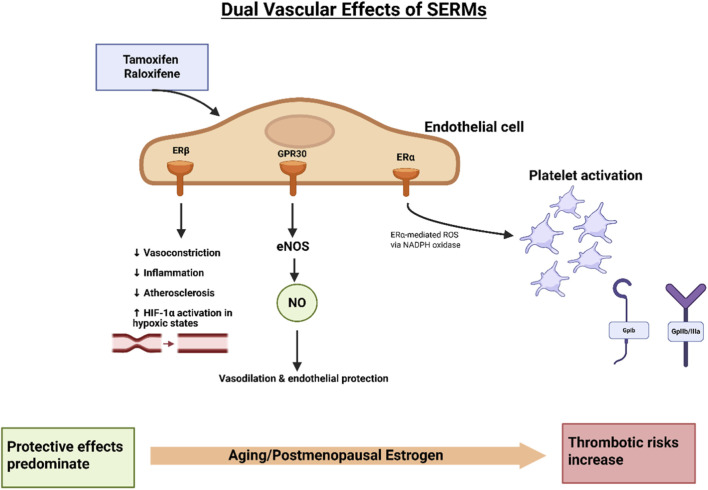
Dual vascular effects of SERMs. Tamoxifen and raloxifene exert both protective and adverse vascular effects through ER signaling in endothelial cells. Binding to ERβ and GPR30 promotes eNOS activation, resulting in NO release, which mediates vasodilation, anti-inflammatory effects, and protection against atherosclerosis. ERβ signaling further reduces vasoconstriction, inflammation, and enhances HIF-1α activity under hypoxic conditions. In contrast, ERα activation can stimulate ROS generation via NADPH oxidase, contributing to platelet activation and prothrombotic effects. The overall balance of vascular protection *versus* thrombotic risk depends on age and hormonal status: protective effects predominate in younger women, whereas aging and postmenopausal estrogen deficiency shift the balance toward increased thrombotic risk (Meyer et al., 2011; da Silva et al., 2021).

While raloxifene and tamoxifen both increase venous thromboembolism (VTE) risk similarly to estrogen, their long-term cardiovascular impact remains incompletely characterized. Both agents induce acute coronary artery relaxation mediated by NO release, promoting vasodilation (Faiz et al., 2021); however, their effectiveness in reducing atherosclerotic plaque burden is limited, as demonstrated in hypercholesterolemic models (Figure 3). Large-scale clinical trials such as STAR and RUTH continue to investigate cardiovascular outcomes associated with SERMs, yet detailed understanding of their effects on venous vasculature, endothelial signaling, and vascular smooth muscle cell function remains insufficient (Castrellon and Gluck, 2008; Barrett-Connor et al., 1998). Notably, the STAR trial revealed that raloxifene reduces thromboembolic events by approximately 30% compared to tamoxifen, particularly lowering risks of PE and deep vein thrombosis (Vogel, 2009). Despite this, both SERMs favorably influence cardiovascular risk markers, including lipid profiles, homocysteine, and C-reactive protein levels, potentially contributing to their vascular protective effects (Wong et al., 2017). The interplay between these benefits and thrombotic risks highlights the complexity of SERM cardiovascular pharmacology and necessitates careful, individualized clinical monitoring. The balance between efficacy and safety is delicate and demands further elucidation through ongoing research.

SERMs robustly enhance endothelial function by stimulating eNOS activation, leading to an increased NO bioavailability and improved vascular tone (Zheng and Houston, 2019). The activation of eNOS occurs through multiple mechanisms, including phosphorylation mediated by kinases such as Akt via ERα-PI3K interactions, as well as calcium/calmodulin binding, both of which promote robust NO production critical for vasodilation (Zahreddine et al., 2020). ERβ further contributes by suppressing vascular inflammation and slowing atherosclerosis progression, while ERα maintains eNOS expression to preserve endothelial resilience, particularly under hypertensive conditions. Additionally, the G-protein-coupled receptor GPR30 augments pulmonary NO synthesis, alleviating right ventricular overload and reducing myocardial fibrosis (Diaz-Zegarra et al., 2024). Together, ERα and ERβ synergistically regulate pulmonary vascular tone, underscoring eNOS activation as a central mechanism through which SERMs confer vascular protection (Tran et al., 2022). This robust endothelial modulation by SERMs is foundational to their cardiovascular benefits.

SERMs, including tamoxifen and raloxifene, intricately affect the coagulation system and platelet function, producing a complex balance between potential antithrombotic and prothrombotic effects (Didembourg et al., 2024). Both agents consistently reduce plasma fibrinogen, a key coagulation glycoprotein, which may lower thrombotic risk (Nabulsi et al., 1993). However, the effects of SERMs on other coagulation factors such as factor VII, antithrombin III, and plasminogen activator inhibitor-1 (PAI-1) remain inconsistent across studies, leaving their overall influence on thrombotic risk unclear. Importantly, despite potential anticoagulant effects, clinical evidence robustly associates SERMs with elevated VTE risk, mirroring the known thrombotic profile of estrogen therapy (Didembourg et al., 2024).

Albeit through distinct mechanisms, both tamoxifen and raloxifene modulate platelet aggregation, a critical step in thrombogenesis. Tamoxifen enhances platelet activation by promoting ROS generation via an NADPH oxidase-dependent pathway, with its active metabolite 4-hydroxy-tamoxifen further amplifying this effect by increasing platelet superoxide release, thereby intensifying pro-thrombotic potential (Johnson et al., 2017). Interestingly, tamoxifen’s influence on platelets is context-dependent, exhibiting both pro- and anti-aggregatory properties depending on the vascular and cellular environment. Raloxifene, in contrast, may exert more favorable vascular effects by not only enhancing NO production, a known inhibitor of platelet aggregation (Polini et al., 2007) but also by directly inhibiting aggregation. This dual action contributes to raloxifene’s overall vascular protective profile (Nanetti et al., 2008). Thus, while both SERMs affect platelet function, raloxifene’s enhancement of NO bioavailability likely confers a more favorable balance between platelet activation and inhibition compared to tamoxifen, highlighting the complex interplay between thrombotic risk and vascular protection inherent in SERM therapy.

In hormone-sensitive breast cancer, platelet activation is further intensified through direct platelet-tumor cell interactions, wherein tamoxifen upregulates platelet adhesion molecules like P-selectin on certain breast cancer cells (Pather et al., 2019). This promotes platelet aggregation and fibrin network formation, contributing to a hypercoagulable state and increased thrombosis risk in patients receiving SERM therapy (Johnson et al., 2017). Altogether, these diverse, context-specific actions of SERMs on coagulation cascades and platelet physiology underline the critical need for individualized risk assessment and vigilant clinical monitoring to balance therapeutic benefits against thrombotic hazards.

From a translational standpoint, the balance between ERα-, ERβ-, and GPR30-mediated signaling emerges as a critical determinant of vascular outcome. While SERMs can enhance nitric oxide bioavailability and attenuate vascular inflammation, concurrent activation of redox-sensitive platelet and coagulation pathways may offset these benefits, offering a mechanistic explanation for the discordance between favorable vascular biomarkers and persistent thromboembolic risk in clinical settings.

## 4 Vascular benefits of SERMs

SERMS exert multifaceted effects on cardiovascular health, extending well beyond their established role in modulating thrombotic risk. Their actions include enhancements of endothelial function, regulation of lipid metabolism, and engagement of broader cardioprotective pathways that may collective influence vascular integrity and disease outcomes (Kang et al., 2022). By promoting NO bioavailability, stabilizing cholesterol levels, and potentially improving systemic metabolic parameters, SERMs emerge as agents capable of reducing atherosclerotic burden and mitigating the risk of coronary artery disease (Christodoulakos et al., 2006). These mechanisms position SERMs at the interface between endocrine modulation and cardiovascular protection, warranting detailed examination of their vascular effects (Zarezade et al., 2023) (Figure 3).

### 4.1 Endothelial function and oxidative stress

One of the most consistent cardiovascular effects of SERMs is the preservation of endothelial homeostasis. Endothelial cells represent the first line of defense against vascular injury, and their dysfunction is a pivotal step in atherogenesis. SERMs, particularly raloxifene, enhance NO production through rapid activation of endothelial eNOS, thereby improving vasodilation and vascular remodeling (Simoncini et al., 2002). NO confers widespread vascular protection by reducing vascular resistance, suppressing platelet aggregation, and attenuating the expression of pro-inflammatory cytokines and adhesion molecules (Cyr et al., 2020). In parallel, SERMs influence VSMC proliferation, apoptosis, and intracellular calcium dynamics, processes central to vascular remodeling and the maintenance of arterial elasticity. These protective effects converge with endogenous estrogen pathways, whereby estrogen reduce adhesion molecule expression and stimulate vascular endothelial growth factor (VEGF) synthesis, thereby facilitating angiogenesis and re-reendothelialization after injury (Trenti et al., 2018).

At a complementary level of intracellular regulation, estrogenic signaling also intersects with cyclic AMP–dependent pathways that shape vascular responsiveness (Jeyaraj et al., 2012). Experimental studies demonstrate that estrogen can activate exchange protein directly activated by cAMP (Epac), leading to increased α2C-adrenergic receptor expression and trafficking in vascular cells (Motawea et al., 2013; Eid et al., 2008). This cAMP-Epac signaling axis has been implicated in stress-induced arteriolar constriction, thus providing a mechanistic link between estrogen signaling and sympathetic vascular control (Slika et al., 2023; Wehbe N. et al., 2020; Eid, 2012). In addition, it may impact endothelial function, mechanisms relevant in postmenopausal vascular regulation.

In addition to NO-mediated pathways, SERMs counteract oxidative stress, a major determinant of endothelial dysfunction and atherosclerotic plaque development. As partial ER agonists within vascular tissues, they upregulate endogenous antioxidant enzymes and inhibit the oxidation of LDL-C, which is critical in preventing lipid-rich plaque formation (Batty et al., 2022). Indeed, evidence demonstrates that raloxifene reduces ROS production, enhances prostacyclin synthesis, and sustains overall endothelial responsiveness (Madamanchi et al., 2005). Taken together, these findings reinforce the concept that SERMs support vascular health not only through acute modulation of vascular tone but also by reducing oxidative injury, thereby conferring long-term protection against atherosclerotic disease. Clinically, disruption of this NO–oxidative stress equilibrium—even in the presence of improved lipid profiles—may accelerate arterial stiffness and microvascular dysfunction, helping to explain why endothelial-level benefits of SERMs do not consistently translate into reduced cardiovascular event rates.

### 4.2 Lipid metabolism and cardiovascular risk

The impact of endocrine therapy on lipid metabolism has become an area of increasing clinical relevance due to its implications for cardiovascular risk (Li et al., 2024). While endocrine therapies for ER–positive breast cancer remain essential to long-term disease control, their potential to alter lipid homeostasis has raised concern, as therapy-induced dyslipidemia can predispose patients to cardiovascular morbidity (He et al., 2020). However, evidence across randomized controlled trials (RCTs), systematic reviews, and meta-analyses has been heterogeneous. A recent network meta-analysis consolidating direct and indirect trial data highlighted the necessity of regular lipid monitoring during endocrine treatment to detect clinically relevant alterations (Zeng et al., 2025).

Tamoxifen provides the clearest example of SERM-mediated lipid modulation, especially in postmenopausal women (Walsh et al., 1991). It consistently reduces total cholesterol (TC) and LDL-C, although its impact on high-density lipoprotein cholesterol (HDL-C) remains variable, with reports describing both increases and neutral effects (He et al., 2021). In the short term, tamoxifen reduces TC, LDL-C, and apolipoprotein B while modestly increasing apolipoprotein A1. Long-term therapy sustains reductions in TC, LDL-C, and lipoprotein(a), alongside preservation or augmentation of apolipoprotein A1, though HDL-C levels often remain unchanged. A clinically important caveat, however, is tamoxifen’s potential to raise triglyceride levels, which in rare cases culminates in severe hypertriglyceridemia (Lewis, 2007). Data from meta-analyses suggest that these lipid-lowering effects are most pronounced with higher drug doses (≥20 mg/day), during shorter treatment durations (≤1 year), and in patients with preexisting dyslipidemia or elevated BMI. In overweight individuals, greater reductions in TC and LDL-C are observed, whereas reductions in HDL-C appear more prominent in breast cancer patients (Alomar et al., 2022).

In premenopausal patients, tamoxifen improves lipid metabolism by lowering TC and LDL-C while increasing HDL-C, effects consistent with its partial estrogen agonist profile (Wang et al., 2022). Notably, combining tamoxifen with ovarian function suppression (OFS) does not markedly alter lipid profiles compared to tamoxifen alone, suggesting that tamoxifen preserves lipid stability under conditions of induced hypoestrogenism (Pagani et al., 2014). Conversely, OFS combined with aromatase inhibitors (AIs) is linked to marked increases in TC and LDL-C, consistent with clinical observations of elevated cardiovascular risk attributable to profound estrogen suppression (He et al., 2021). From a cardiometabolic standpoint, tamoxifen therefore appears to provide a more favorable balance compared to AIs, particularly in patients at elevated cardiovascular risk. When dyslipidemia arises during endocrine therapy, individualized management that includes lifestyle interventions and, where appropriate, statin therapy is recommended (Wang et al., 2022). Collectively, these findings emphasize the value of tailoring endocrine strategies and underscore the importance of regular lipid profile monitoring in patients undergoing long-term hormonal treatment for breast cancer.

### 4.3 Therapeutic considerations

The intricate interplay between ER subtypes offers an opportunity for receptor-selective targeting in cardiovascular therapy. ERα has emerged as the key mediator of vasodilatory and reparative responses, including eNOS activation, NO production, and endothelial regeneration, which together contribute to vascular protection (Darblade et al., 2002). By contrast, ERβ may counteract some of these salutary effects. Studies demonstrate that the absence of ERβ enhances estradiol-induced vasorelaxation, suggesting an inhibitory role for this receptor in vascular physiology (Elsaid et al., 2025). Accordingly, future therapeutic approaches may require strategies that preferentially engage ERα activity while limiting ERβ activation to optimize vascular outcomes.

SERMs exemplify this principle by selectively mimicking beneficial estrogen effects in the vasculature while minimizing systemic adverse outcomes. Raloxifene, in particular, has demonstrated multifaceted cardiovascular actions, including acute vasodilation, inhibition of vascular smooth muscle proliferation, attenuation of myocardial hypertrophy, and improved coronary perfusion, all of which are mediated through NO release and related endothelial mechanisms (Nakamura et al., 2004). Their partial estrogen agonist activity permits cardiovascular protection without recapitulating the full spectrum of estrogen’s endocrine effects, representing an attractive therapeutic model for modulating cardiovascular risk.

### 4.4 SERMS, vascular aging, and endothelial senescence

Vascular aging is characterized by progressive endothelial dysfunction, increased oxidative stress, telomere attrition, and the accumulation of senescent endothelial and VSMCs, all of which contribute to heightened cardiovascular vulnerability in postmenopausal women (Bloom et al., 2023; Bendale et al., 2013). Estrogen signaling plays a central role in modulating these processes (Bendale et al., 2013). Indeed, loss of estrogenic tone after menopause accelerates endothelial senescence through impaired NO bioavailability, mitochondrial dysfunction, and activation of proinflammatory pathways (Moreau et al., 2020). By selectively engaging ER–dependent signaling, SERMs have the potential to partially modulate age-associated vascular decline, although their effects appear to be highly context- and receptor subtype–dependent.

Preclinical studies suggest that ERα and ERβ activation can differentially influence cellular senescence pathways, including regulation of oxidative stress responses, autophagy, and cell-cycle arrest (Xiang et al., 2023; Ambhore et al., 2018; Song et al., 2020). In vascular endothelial cells, SERMs have been shown to preserve NO signaling and attenuate oxidative injury, mechanisms that may delay functional aspects of vascular aging (Wong et al., 2008; Sasaki et al., 2020). However, these putative anti-senescent effects coexist with age-related shifts in hemostatic balance and endothelial–platelet interactions, underscoring the complexity of SERM actions in older vascular systems. Collectively, these observations suggest that age and senescence burden may critically modify vascular responses to SERMs, reinforcing the need to consider biological aging, not chronological age alone, when evaluating cardiovascular risk and benefit in postmenopausal populations.

## 5 Vascular risks associated with SERMs

Despite their vascular benefits, the clinical use of SERMs is complicated by significant cardiovascular risks, most notably an increased incidence of venous and arterial thromboembolic events (Jia et al., 2025). Importantly, improvements in lipid-lipoprotein profiles observed with certain SERMs do not consistently translate into lower CVD incidence, underscoring the complexity of their net vascular effects (Bush et al., 2001). In practice, potential cardiometabolic advantages may be counterbalanced by pro-thrombotic properties, ischemic complications, and inconsistencies in endothelial function modulation (Blondon et al., 2022). This duality requires careful contextualization of SERM use in patients with varying baseline cardiovascular risk.

Thromboembolic complications remain the most important vascular liability associated with SERMs. Conditions such as deep vein thrombosis and PE are consistently reported, and risk amplification is particularly evident in populations with predisposing factors including prolonged immobility, smoking, or prior thrombotic events (Park and Jordan, 2002). From a mechanistic perspective, SERMs such as tamoxifen and raloxifene promote a hypercoagulable state by upregulating procoagulant factors and downregulating natural anticoagulant pathways, thereby disturbing hemostatic balance (Lin et al., 2018; Cosman et al., 2005; Nordstrom et al., 2022). Beyond venous thrombosis, tamoxifen use has also been linked to ischemic stroke, transient ischemic attacks and retinal vein thrombosis, with the highest risks noted in long-term users (Bushnell and Goldstein, 2004; Lai et al., 2017; Ghanavati et al., 2023). A pivotal randomized placebo-controlled trial involving over 10,000 postmenopausal women found that raloxifene increased the risk of VTE and fatal stroke, with smoking status modifying outcomes (Mosca et al., 2009). Taken together, these findings indicate that the thromboembolic liability of SERMs is not uniform but is shaped by both drug properties and patient-specific predispositions, underscoring the need for careful clinical stratification.

The influence of SERMs on endothelial function is complex and sometimes contradictory. Mechanistic studies suggest beneficial modulation of biomarkers, including improvement in the NO to endothelin-1 ratio, lowering of C-reactive protein and homocysteine, and reduction of inflammatory cytokines such as TNF-α and IL-6 (Walsh et al., 2000; Bonanni et al., 2003; Leung et al., 2007; Gianni et al., 2004). Despite these favorable biochemical trends, large-scale clinical trials fail to demonstrate consistent reductions in cardiovascular events (Christodoulakos et al., 2006; Lamas et al., 2015). This discrepancy highlights the translational gap between surrogate vascular endpoints and hard cardiovascular outcomes, emphasizing that mechanistic improvements do not always equate to clinical benefit. Consequently, enthusiasm for widespread SERM use in cardiovascular prevention remains tempered by this uncertainty.

Concerns about vascular risk also contribute to hesitancy in the preventive use of SERMs, particularly tamoxifen. Although tamoxifen effectively reduces breast cancer incidence, its uptake is suboptimal, particularly among younger women under 50, largely because of safety concerns (Bryce, 1998; Jayasekera et al., 2023). Evidence shows that physician recommendations significantly influence patient acceptance, suggesting that enhanced counseling regarding both risks—such as VTE, PE, and endometrial cancer—and protective benefits could improve adoption. Presently, tamoxifen and raloxifene remain the only FDA-approved chemo-preventive agents for premenopausal women (Kaplan et al., 2012), while newer SERMs and aromatase inhibitors are mainly assessed in postmenopausal settings (Generali et al., 2023). These dynamics illustrate how perceptions of vascular risk powerfully shape therapeutic decisions, sometimes diminishing the real-world implementation of effective preventive strategies.

Emerging studies point toward dose and duration as modifiable determinants of vascular safety. Low-dose, short-duration tamoxifen regimens have demonstrated the ability to retain chemo-preventive efficacy while reducing thromboembolic and endometrial risks (Buijs et al., 2024; Emons et al., 2020). Such regimens may offer a strategy to balance efficacy with improved safety, potentially lowering the incidence of secondary cancers and thromboembolic events. Nevertheless, these findings require validation in prospective long-term trials to ensure oncologic effectiveness while confirming vascular protection (Iqbal et al., 2012). The pursuit of optimized dosing highlights a central theme: tailoring endocrine therapy to maximize benefit while minimizing harm.

Comparison with hormone replacement therapy (HRT) provides additional perspective on relative vascular risk. Unlike SERMs, which act as antagonists in breast tissue and partial agonists in vascular and skeletal tissues (Lewis and Jordan, 2005), HRT exposes patients to systemic estrogen—often combined with progestins—that increases blood pressure, alters lipid metabolism, augments inflammation, and destabilizes vascular plaques (Johansson et al., 2022; Nie et al., 2022; Yang et al., 2013). These mechanisms translate into higher rates of myocardial infarction and stroke with HRT use (Gialeraki et al., 2018; Henderson and Lobo, 2012). A meta-analysis demonstrated that HRT raised stroke risk by 32% and VTE risk by 105%, although coronary artery disease was not significantly elevated, thereby emphasizing the stronger vascular risk profile of HRT compared with SERMs (Artero et al., 2012). This contrast highlights that while SERMs are not devoid of vascular hazards, they represent a safer alternative to systemic hormone replacement, especially in populations requiring long-term endocrine modulation. Taken together, these findings highlight that molecular indicators of coagulation or endothelial improvement alone are insufficient predictors of vascular safety, reinforcing the need for integrated risk stratification when SERMs are prescribed in populations with heterogeneous cardiovascular vulnerability.

## 6 Insights from major clinical trials

The relationship between SERMs and cardiovascular outcomes remains incompletely defined, largely due to the varying influence of individual patient characteristics, concomitant therapies, and baseline risk factors. Two major clinical trials, the STAR (NSABP P-1 and P-2) and RUTH studies, have provided the most robust data regarding the cardiovascular safety of tamoxifen and raloxifene. The STAR trial demonstrated that although both agents significantly reduced breast cancer incidence, tamoxifen use was accompanied by higher rates of thrombotic and embolic complications (Vogel et al., 2010). Similarly, the RUTH trial identified VTE as the most prominent cardiovascular risk, reporting a twofold increase in thrombotic events with tamoxifen, while raloxifene carried a comparatively lower risk (Mosca et al., 2009). These findings suggest that raloxifene may offer a safer vascular profile than tamoxifen, although both drugs still require judicious patient selection and vigilant monitoring to minimize adverse cardiovascular outcomes.

Although SERMs enhance endothelial nitric oxide bioavailability and attenuate vascular smooth muscle cell hyperplasia, this mechanistic vasculoprotection contrasts with a net prothrombotic hemostatic perturbation, as reflected by persistently elevated risks of pulmonary embolism and deep vein thrombosis. Despite mechanistic evidence supporting vascular health benefits, including attenuation of atherosclerosis progression and improvements in lipid metabolism (Choi et al., 2008; Westphal et al., 2012), SERMs have not consistently demonstrated reductions in hard cardiovascular endpoints such as myocardial infarction or cardiovascular mortality. Their favorable biochemical and surrogate effects therefore do not substitute for established therapies like statins, antiplatelet agents, or antihypertensive drugs (Herrington and Klein, 2001). In clinical practice, careful patient evaluation is indispensable, with age, baseline cardiac status, and comorbidities serving as critical determinants of therapy-associated cardiovascular risks. This reinforces that SERMs should not be positioned as cardioprotective agents but rather as selective endocrine therapies in which cardiovascular safety must be actively weighed against oncologic benefits. This unresolved paradox underscores the need for subtype-specific clinical trials that prioritize ERβ-selective agonism over mixed receptor profiles, with cardiovascular safety—rather than surrogate vascular markers—serving as a primary endpoint.

Data from large trials including MORE and RUTH trials further identify age as a meaningful modifier of cardiovascular risk among SERM users. Evidence indicates that younger postmenopausal derive more favorable cardiovascular safety profiles with raloxifene compared to older women treated with the same agent (Vogelvang et al., 2006). Likewise, differential risk–benefit considerations must guide SERM choice: tamoxifen remains a potent chemo-preventive agent but carries higher risks of thromboembolic events and endometrial cancer, whereas raloxifene offers a comparatively safer gynecologic and vascular profile (Patricio et al., 2013). These clinical contrasts underscore the necessity of aligning drug selection not only with oncologic prevention but also with an individualized assessment of cardiovascular vulnerability, ensuring that the therapeutic strategy balances efficacy with safety.

## 7 Research gaps and future perspectives

Patient-customized risk assessment models are essential for optimizing SERM therapy by judiciously balancing therapeutic efficacy against the potential for cardiovascular risk. Incorporating genetic markers, including variants such as *Factor V* Leiden and *CYP2D6*, alongside clinical biomarkers like D-dimer, hs-CRP, and fibrinogen will refine our capacity to predict drug metabolism and individual risk for thromboembolic events (Taylor et al., 2020; Petricevic et al., 2015). Innovative strategies, including the application of artificial intelligence algorithms that integrate clinical, demographic, and estrogen receptor expression data, promise to elevate the precision of personalized SERM management. In addition, cardiac risk stratification via coronary artery calcium scoring, as per contemporary AHA/ACC guidelines, further enables evidence-based decision-making for populations with prior cardiovascular histories (Dzaye et al., 2019).

A holistic approach integrates pharmacological, lifestyle, and risk-modifying interventions to mitigate adverse vascular outcomes. For at-risk individuals, anticoagulant or antiplatelet prophylaxis may provide added safety, while adjunctive therapies such as statins and metformin can address coexisting metabolic derangements. Individualized monitoring of coagulation and lipid indices, diligent avoidance of modifiable vascular risk factors including smoking and sedentary behavior, and minimizing use of NSAIDs remain paramount (Patricio et al., 2013). Importantly, nutrition and physical activity represent accessible tools to enhance vascular health and minimize thrombotic risk—a reminder that the foundation of cardiovascular prevention remains rooted in the promotion of healthy living.

The trajectory of SERM pharmacology is increasingly defined by the emergence of newer molecules with improved selectivity and safety profiles, such as next-generation SERMs and selective estrogen receptor degraders. New agents such as bazedoxifene and ospemifene demonstrate a favorable reduction in thrombotic and endometrial risk (Ellis et al., 2015). There is also a growing emphasis on ERβ-targeted agents, whose vasculoprotective properties provide a complement to the more complex roles of ERα. Notably, ERα-selective agonists like 16α-LE2 appear to improve endothelial function and prevent cardiac hypertrophy in hypertensive models (Bush et al., 2001), while ERβ agonists mediate vasodilation and downregulate angiotensin-converting enzyme (Cano et al., 2007). Nonetheless, raloxifene persists as a benchmark for safety and efficacy in the prevention of osteoporosis and breast cancer, surpassing the long-term profiles of tamoxifen (Vogelvang et al., 2006).

Emerging therapeutic strategies that combine nitric oxide donors with anti-inflammatory agents offer promise for enhancing vascular protection while reducing thrombotic risk. Nitric oxide donors improve endothelial function by promoting vasodilation and inhibiting platelet aggregation, complementing the selective actions of SERMs. Meanwhile, selective estrogen receptor degraders (SERDs) represent a novel therapeutic class that achieves receptor degradation rather than modulation, potentially minimizing cardiovascular side effects associated with endocrine therapy. Although SERDs may theoretically avoid partial agonist signaling implicated in SERM-related thrombotic risk, their impact on endothelial function and metabolic cardiovascular protection remains poorly defined, and they should currently be viewed as comparative tools to inform receptor-specific vascular biology rather than as substitutes for SERMs in postmenopausal cardiovascular care. Together, these advances provide a promising framework to optimize cardiovascular safety and efficacy in hormone-based treatments (Abdel-Magid, 2017).

It is clear that optimizing outcomes would require collaboration between cardiology and oncology specialists. Indeed, such joint decision-making is crucial given SERMs’ multifaceted effects on vascular function (Regitz-Zagrosek et al., 2007). Moreover, pediatric cancer survivors require routine cardiovascular monitoring, including echocardiography and vascular assessments (Dillenburg et al., 2013). Ongoing research into non-hormonal preventive options and combination regimens seeks to enhance therapeutic efficacy and safety. By combining precise risk stratification, close monitoring, lifestyle interventions, pharmacologic measures, and emerging therapeutics within an integrated multidisciplinary framework, SERM use can become progressively more personalized, safe, and effective.

Despite substantial advances in understanding SERM biology, several critical research gaps remain. In particular, adequately powered cardiovascular endpoint trials evaluating newer or ERβ-selective SERMs are lacking, limiting definitive conclusions regarding long-term vascular safety. In addition, the feasibility and benefit of combined therapeutic strategies—such as concurrent SERM use with antithrombotic or endothelial-protective agents—have not been systematically examined in prospective studies. Addressing these gaps will be essential to determine whether selective receptor targeting or adjunctive approaches can preserve therapeutic efficacy while mitigating thrombotic risk in postmenopausal women.

## 8 Conclusion

SERMs represent a clinically valuable yet inherently paradoxical class of endocrine therapies, offering measurable vascular and metabolic benefits while simultaneously conferring an increased risk of thrombotic events. Their ability to enhance endothelial function, favorably modulate lipid metabolism, and attenuate certain inflammatory pathways contrasts with a well-documented propensity to promote venous and, in some contexts, arterial thrombosis. This duality defines the central clinical challenge of SERM therapy, particularly in postmenopausal women, in whom cardiovascular risk is already heightened by age, hormonal changes, and comorbidities.

From a practical standpoint, SERMs should not be viewed as cardioprotective agents but rather as endocrine therapies whose cardiovascular effects must be actively weighed during clinical decision-making. Optimal use requires individualized assessment of baseline thrombotic risk, vascular health, and competing oncologic or skeletal priorities. In postmenopausal cardiovascular care, this balance underscores the need for careful patient selection, vigilant monitoring for thrombotic signals, and integration of SERMs within a broader risk-reduction framework that includes lifestyle modification and standard cardioprotective therapies. Ultimately, appreciating and managing this benefit–risk equilibrium is essential to maximizing the clinical utility of SERMs while minimizing preventable vascular harm.

As detailed in Section 4, decoupling SERM-mediated platelet NADPH oxidase activation from beneficial lipid and endothelial modulation—potentially through novel coregulator-targeted or subtype-selective agents—represents a paramount developmental priority. Future efforts should prioritize cardiovascular endpoint trials of ERβ-selective SERMs in postmenopausal women at elevated ASCVD risk, alongside prospective evaluation of combined SERM–antithrombotic strategies. Importantly, translational studies must also validate pragmatic risk-stratification tools—including pharmacogenomic triage such as *CYP2D6* genotyping and longitudinal D-dimer screening—to better define SERM candidacy. Such approaches may enable mitigation of the approximately 1.5–2-fold excess venous thromboembolism risk associated with SERM therapy while preserving potential vascular and metabolic benefits, thereby advancing safer and more individualized cardiovascular care.
